# Timely Diagnosis of Histoplasmosis in Non-endemic Countries: A Laboratory Challenge

**DOI:** 10.3389/fmicb.2020.00467

**Published:** 2020-03-24

**Authors:** María José Buitrago, M. Teresa Martín-Gómez

**Affiliations:** ^1^Mycology Reference Laboratory, National Centre of Microbiology, Instituto de Salud Carlos III, Madrid, Spain; ^2^Microbiology Department, Vall d’Hebron University Hospital, Barcelona, Spain

**Keywords:** histoplasmosis, laboratory, diagnosis, non-endemic areas, PCR

## Abstract

Human histoplasmosis is a fungal infection caused by the inhalation of microconidia of the thermally dimorphic fungi *Histoplasma capsulatum*. Autochthonous cases of histoplasmosis have been diagnosed in almost every country, but it is considered an endemic infection in specific areas of the world. Many of them are popular travel destinations or the source of migratory movements. Thus, the vast majority of the registered cases in non-endemic countries are imported. They correspond to people having been exposed to the fungus in endemic locations as immigrants, expatriates, transient workers or tourists, with reported cases also associated to organ donation. Misdiagnosis and delays in initiation of treatment are not uncommon in cases of imported histoplasmosis. They are associated to high fatality-rates specially in patients with compromised cellular immunity in which progressive disseminated forms develop. The diagnosis of this infection in non-endemic countries is hampered by the lack of clinical suspicion and a dearth of available diagnostic tools adequate to offer rapid and accurate results. Non-culture-based assays such as nucleic-acid amplification tests present as a suitable alternative in this situation, offering improved sensitivity and specificity, shortened turnaround time, and increased biosafety by avoiding culture manipulation. In non-endemic regions, molecular techniques are being used mainly in laboratories from countries that have registered an increase in the incidence of imported cases. However, the number of published techniques is limited and lack consensus. Efforts are currently under way to standardize nucleic acid amplification-based techniques for its implementation in areas registering a rising number of imported cases.

We are living an era of massive population movements due to immigration, volunteering, and affordable adventure travels to areas not previously accessible to the general public. This opens the door to the emergence of exotic infections in countries where such illnesses are infrequent (Barboza and Ouatresous, 2007; Schlagenhauf et al., 2015). That is the case of histoplasmosis (Manfredi et al., 1994; Bahr et al., 2015).

Human histoplasmosis is a fungal infection caused by inhalation of microconidia of the thermally dimorphic fungi *Histoplasma capsulatum* v. *capsulatum* and *H. capsulatum* v. *duboisii*. In immunocompetent individuals, exposure to this fungus usually remains unnoticed or manifests as a flu-like respiratory episode, whereas immunocompromised patients are exposed to life-threatening disseminated infections (Wheat et al., 2016).

The distribution of *H. capsulatum* v. *duboisii* seem to be restricted to Sub-Saharian Africa. *H. capsulatum* v. *capsulatum*, in contrast, can be found irregularly distributed worldwide. The latter is more prevalent in the Eastern Coast of United States, Central-America, Northern countries of South-America, South-Eastern Asia, and territories crossed by the Yangtze River and the Brahmaputra River, and is rarely found in more temperate regions of the world (Antinori, 2014; Baker et al., 2019). Thus, although autochthonous cases of histoplasmosis have been described in many countries, it is commonly considered an “endemic infection” only in specific areas, many of which are popular travel destinations (UNWTO, 2019) or the source of migratory movements (International Organization for Migration, 2018).

## Histoplasmosis in Non-Endemic Countries: the Extent of the Problem

Histoplasmosis is not a notifiable disease, and it is not included in most Public Health surveillance systems, making difficult to quantify its real burden (Nacher et al., 2018). Incomplete data exist on the incidence and prevalence of this infection in non-endemic countries. In Spain, a prevalence of 0.31 cases (SD 0.1) per 100,000 population-year has been reported, but this may be an underestimation, as only patients seeking for medical advice upon arrival from a presumed endemic region were included (Molina-Morant et al., 2018). Similar studies are lacking in other non-endemic regions. Available figures indicate that histoplasmosis is the most frequent imported mycosis (Salzer et al., 2018): cases correspond to immigrants, expatriates, transient workers or tourists having had *Histoplasma* exposure in endemic areas (Molina-Morant et al., 2018; Salzer et al., 2018; Staffolani et al., 2018). Transmission related to solid organ donation has been sporadically described (Kamei et al., 2003; Ashbee et al., 2008; Berger, 2018).

On the basis of histoplasmin skin test studies, it has been estimated that up to 20% of travelers returning from Latin America may have had contact with *Histoplasma* (Norman et al., 2009). Activities related to cave exploration, or exposure to soils enriched in nitrogen from bats and birds droppings are the common denominator of up to 29.4% cases of imported histoplasmosis (Kamei et al., 2003; Ashbee et al., 2008). Clusters of cases are associated to groups undergoing leisure or professional activities in high risk locations (Alonso et al., 2007; Cottle et al., 2013), and sum up to 56.2% of well-documented published cases in European travelers (Staffolani et al., 2018).

Spain along with France and Italy have reported the largest numbers of sporadic cases involving travelers and immigrants (Loulergue et al., 2007; Peigne et al., 2011; Buitrago and Cuenca-Estrella, 2012; Nacher et al., 2018; Staffolani et al., 2018). They accumulate up to 64.1% of the cases diagnosed in immunocompetent European travelers (Staffolani et al., 2018), and concentrate 66.4% of travel-related cases belonging to a cluster. Isolated cases have been communicated in several other European countries (Ashbee et al., 2008; Doleschal et al., 2016; Lindner et al., 2018) as well as in Asia (Cho et al., 2018) and the MiddleEast (Segel et al., 2015).

## Clinical Presentation

Most histoplasmosis cases detected in areas of low-endemicity occur following three main patterns. The most difficult to detect corresponds to immunocompetent individuals exposed to a low infectious inoculum that experience an asymptomatic seroconversion or a mild flu-like respiratory episode. This is presumedly the most frequent form of imported histoplasmosis (Norman et al., 2009). After return to their country of origin, these cases usually remain unnoticed unless patients are investigated in the setting of an outbreak (Staffolani et al., 2018). Some patients fail to clear the infection and evolve inadvertently to a chronic form with pulmonary nodules. They may be incidentally found later in life, being commonly misdiagnosed as lung cancer or tuberculosis (Ashbee et al., 2008; Norman et al., 2009; Wheat et al., 2016; Azar et al., 2018; Oladele et al., 2018). Because of the wide time gap between primary exposure and diagnosis, it is hard to establish an epidemiological link, of help to guide medical interventions.

The second pattern is seen in cases of massive *Histoplasma* exposure (i.e., high-risk activities in heavily contaminated areas), after which immunocompetent patients may develop an acute pneumonia days to a few weeks later. This presentation was reported in 90.7% of the cases included in the Staffolani’s review (Staffolani et al., 2018).

The third pattern corresponds to progressive disseminated histoplasmosis, usually seen in patients with compromised cellular immunity, mainly related to HIV infection, but also to organ transplant and biological therapies, in particular anti-TNF-α drugs (Baddley et al., 2018). This scenario is frequently associated to individuals that have resided for long periods of time in endemic areas before moving to receptor countries, but and manifests in the setting of an acquired immunologic incompetence (Ashbee et al., 2008; Norman et al., 2009).

Risk of reactivation exists even decades after the initial infection (Loulergue et al., 2007; Richaud et al., 2014). Up to 25% of cases registered in European travelers developed 5 years after exposure, and 41% of disseminated forms were reactivations of infections occurring at least 5 years before (Ashbee et al., 2008). This event is usually related to an acquired immunocompromise.

The mortality rate of cases diagnosed in non-endemic areas depends on the clinical form, and the immune status of the host, but also on the diagnostic and therapeutic promptness. For immunocompetent individuals it ranges between 2 and 17.4% (Kamei et al., 2003; Molina-Morant et al., 2018; Salzer et al., 2018). A 10% attributable mortality is reported for histoplasmosis in solid organ transplant recipients (Gajurel et al., 2017). The highest fatality rates are associated to misdiagnosed forms of progressive disseminated histoplasmosis: 42% mortality if treatment is delayed, and 100% if antifungal therapy is not prescribed (Ashbee et al., 2008; Scheel et al., 2014). Some cases are only revealed at autopsy (Kamei et al., 2003; Antinori et al., 2006; Denning, 2016).

## Handicaps of Laboratory Diagnosis in Non-Endemic Areas

The two main diagnostic handicaps in non-endemic areas are the usually low index of suspicion, and the scarceness of tools for a fast and accurate identification of the infection.

**FIGURE 1:**
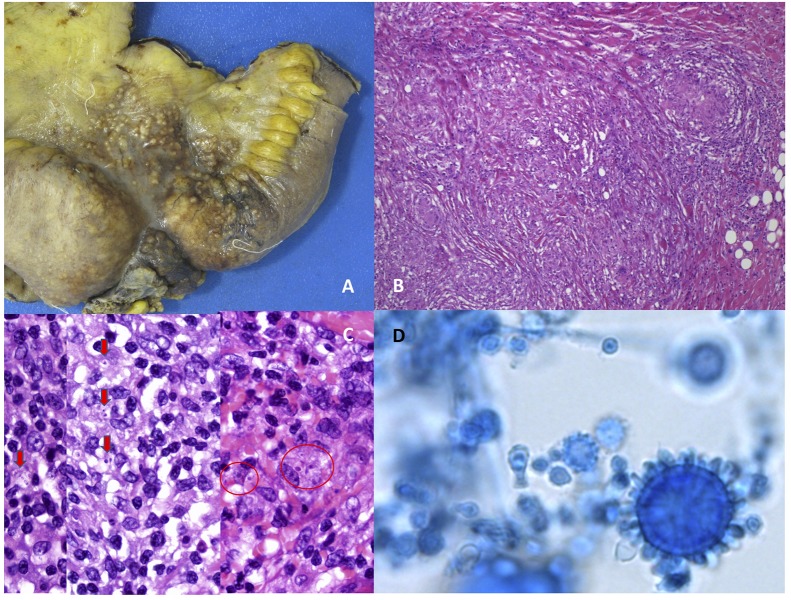
Morphological appearance of *Histoplasma capsulatum* var. *capsulatum* in culture and in affected tissues. Images **(A–C)** are courtesy of Dr. Steffania Landolfi. **(A)** Surgically excised ileum showing stenosis and multiple macroscopic nodules from a case of disseminated histoplasmosis with intestinal involvement. **(B)** Tipical appearance of granulomas in tissue, hematoxilin-Eosin. **(C)** Intracellular yeasts surrounded by a clear area resembling a pseudocapsula (Hematoxilin-Eosin staining). **(D)** Tuberculate macroconidia of *Histoplasma capsulatum* var. *capsulatum* in culture.

Conventional laboratory tests for the diagnosis of histoplasmosis include mycological cultures and histopathology of affected organs and tissues (Figure 1). Isolation of the fungus in culture is considered the gold standard for diagnosis. *H. capsulatum* can grow in mycological media of common use in routine laboratories, and current MALDI-TOF systems can provide a presumptive identification (Panda et al., 2015; Rizzato et al., 2015; Rychert et al., 2018; Valero et al., 2018). Cultures, however, have well-known limitations, i.e., slowness, suboptimal sensitivity, and requirement of BSL3 facilities to manipulate them (Kauffman, 2009). Typical tuberculate macroconidia closely resemble the saprophyte *Sepedonium*, and microconidia can be misidentified as *Chrysosporium*. The histopathological observation also requires skilled personnel. A plethora of findings complicates the microscopic diagnosis to pathologists non-familiar with this infection: images range from localized granulomas to extensive aggregates of macrophages fulfilled with small yeasts surrounded by pseudocapsules (Woods and Schnadig, 2003). Intracellular yeasts are often confused with *Leishmania* spp., *Cryptococcus* or *C. glabrata* by non-expert observers (Wheat, 2003; Wheat et al., 2016).

Complement fixation (CF) or Immunodiffusion (ID) are common techniques used for *Histoplasma* antibody detection. Their sensitivity greatly varies depending on the clinical form and the immune status of the host. Antibody detection is of limited usefulness in severely immunocompromised individuals, and to diagnose acute infections in early phases (Azar and Hage, 2017). In non-endemic areas serology is usually performed in reference centers as a complementary tool, meaning that results are not readily available. Despite this, antibody detection was used in the diagnostic workup of 74.7% cases reported in immunocompetent European travelers (Staffolani et al., 2018). Novel FDA cleared tests seem to be of help in the differential diagnosis of pulmonary nodules, but currently they are not available overseas (Deppen et al., 2019).

*Histoplasma* antigen detection in body fluids is currently considered the most sensitive and quickest technique to diagnose this infection (Azar and Hage, 2017). It is particularly useful in progressive disseminated forms, and it has proven its applicability to infected animals that may act as potential reservoirs for humans (Cunningham et al., 2015; Rothenburg et al., 2019). Combined detection in serum and urine seem to provide the best diagnostic performance, with some differences in the diagnostic yield between FDA cleared tests (Theel et al., 2013; Fandiño-Devia et al., 2016; Azar and Hage, 2017; Torres-González et al., 2018). Antigen detection provides a clear improvement for the management of histoplasmosis, especially in highly endemic areas where this infection frequently coexists with tuberculosis (Bansal et al., 2019), and recently it has been included in the WHO list of essential diagnostic test^1^. Despite FDA-approved commercial tests are available, their use is mostly restricted to developed endemic areas (Bongomin et al., 2019a). There is little data regarding its performance in non-endemic areas. The reported use of antigen detection in non-United States cases of imported histoplasmosis is very limited: performance of an antigen test (in urine and/or serum) was declared only in 9 out of 319 cases of histoplasmosis in immunocompetent travelers diagnosed out of the United States (Staffolani et al., 2018). Even in non-endemic areas of the United States, the use of the EIA antigen detection has been seldom reported (Benedict et al., 2015). The underlying reason may be at least partially related to the overall scarcity of imported cases, making the test non-cost-effective in settings of no endemicity.

## Molecular Alternatives to Diagnose Histoplasmosis

Historically, antigen detection tests use to be not available in non-endemic regions, so molecular techniques may represent a suitable alternative for a rapid diagnosis. They offer the opportunity to shorten turnaround times, and to diminish the risk of laboratory-acquired infection where laboratory personnel is not used to handle *Histoplasma* positive cultures. In non-endemic regions, the use of molecular techniques has been described mainly in countries that have registered an increase in the incidence of imported cases, such as Spain (Buitrago et al., 2011). Staffolani reflected the sporadic use of PCR techniques in travelers (Staffolani et al., 2018) and, more recently, a case was diagnosed with the use of a panfungal assay in Germany (Lindner et al., 2018). Beside this, PCR-based techniques have been claimed to be of help in the detection of environmental reservoirs, and in the design of strategies of intervention to prevent exposure risk (Gómez et al., 2018, 2019).

No commercial PCR-based test has been approved for *in vitro* diagnosis yet, but published techniques show promising results. In a recent meta-analysis Caceres and coworkers reported an overall sensitivity and specificity (95% CI) of 95.4 (88.8–101.9) and 98.7 (95.7–101.7) respectively in cases of progressive disseminated histoplasmosis of HIV + patients (Caceres et al., 2019). To date, the reported assays target different regions in the genome, being the most successful the ITS multicopy region of the ribosomal DNA, and genes encoding the M antigen or the 100-kDa-like protein. Techniques encompass conventional and nested PCR, as well as the more user-friendly and less cumbersome real-time PCR (table 1). A Reference Laboratory in Spain has developed various real time PCR-based assays, showing an excellent performance for the diagnosis of 39 cases of histoplasmosis (Buitrago et al., 2006, 2011; Gago et al., 2014). Samples were obtained from cases of probable infection in immunocompetent travelers (23%), and of proven histoplasmosis diagnosed in immigrants (77%), 97% of them having AIDS as underlying disease. The sensitivity of the PCR for disseminated disease was 89% showing superiority over mycological culture (73%) and antibody detection (40%) (Buitrago et al., 2011). An interesting multiplex approach developed by the same group targets mixed infections with *Pneumocystis jirovecii* and *Cryptococcus neoformans*, common opportunistic fungal pathogens of HIV-infected patients. This design showed an overall sensitivity of 93 and 100% specificity.

**TABLE 1:** Characteristics of published nucleic acid amplification assays based on conventional and Real Time PCR for the diagnosis of histoplasmosis.

References	Country	PCR assay	Target	Clinical samples	Sensitivity (samples tested)	Specificity
Bialek et al., 2001*	Germany/United States	Conventional (nested)	18S rDNA	Blood, spleen, lung (mice)	83.1%	ND
Rickerts et al., 2002	Germany/United States	Conventional (nested)	100-kDa-like protein Gene	Biopsy	70%	100%
Guedes et al., 2003^1^	Brazil	Conventional	M antigen gene	NO^1^	100%	100%
Bracca et al., 2003	Argentina	Conventional (semi-nested)	M antigen gene	Biopsy, blood, mucose	ND (30)	ND
Martagon-Villamil et al., 2003^2^	United States	Real time	ITS rDNA	BAL, lung biopsy, bone marrow	100% (3)	100%
Maubon et al., 2007	French Guiana	Bialek et al., 2002	100-kDa-like protein Gene	Blood, serum, BAL, BAS, biopsy, CSF, others	100% (40)	100%
Buitrago et al., 2011	Spain	Real time	ITS rDNA	Blood, serum, bone marrow, sputum, BAS, BAL, biopsy, CSF, others	89% Proven H (54); 60% probable H (13)	100%
Simon et al., 2010	French Guiana	Real time	ITS rDNA	BAL, biopsy, bone marrow, CSF	95,4% (348)	96%
Gago et al., 2014	Spain	Multiplex real time	ITS rDNA	BAL, biopsy, serum, bone marrow	92.5% (72)	100%
López et al., 2017^3^*	Colombia	Real time PCR	M antigen gene H antigen gene ITS rDNA	Lung biopsy (mice)	ND	ND^3^

*Murine model; (1) tested on DNA from strains; (2) tested mainly on DNA from strains; (3) comparative analysis of three techniques; BAL, bronchoalveolar lavage; BAS, broncho aspirate; Proven H, proven histoplamosis by EORTC/MSG criteria; Probable H, probable histoplasmosis by EORTC/MSG criteria; ND, no data.

Regarding the best samples to test, different types of specimens have been studied, including respiratory secretions, biopsies, bone marrow, blood, or sera. Good performance of respiratory samples and biopsies has been reported (Buitrago et al., 2011). The sensitivity for blood and bone marrow specimens reached 100% in immunocompromised patients with disseminated disease (Maubon et al., 2007), but was more modest in immunocompetent patients (Buitrago et al., 2011) reflecting the lower amount of circulating DNA circulating in these patients. An important point was the increased sensitivity obtained by testing more than one sample per patient in cases with extra-pulmonary involvement (Gago et al., 2014). Overall, although the diagnostic yield seem variable depending on the disease stage, clinical form, and type of specimen, PCR based techniques may be the answer to provide the much desired rapid results, particularly to diagnose severe cases in non-endemic locations (Vasconcellos et al., 2019).

As compared to other PCR modalities, isothermal nucleic acid amplification techniques are considered cheaper and more user-friendly. No sophisticated equipment is required, and handling can be done by personnel lacking expertise in molecular test (Lee, 2017). However, limited attempts have been made to design isothermal assays. All of them dealt with progressive disseminated histoplasmosis samples of HIV + patients from endemic countries, with reasonably good results (Scheel et al., 2014; Zatti et al., 2019). Their diagnostic yield varied greatly depending on the type of sample tested: an 83% sensitivity was reported for blood and bone marrow as compared to a nested PCR targeting the *Hcp100* gene (Zatti et al., 2019), whereas a more modest 67% sensitivity was achieved in antigen positive urine samples. Altogether, isothermal assays could become suitable for use as a complementary diagnostic test in low-income countries, and potentially useful techniques for implementation in non-endemic areas.

Major drawbacks of in-house molecular tests are the lack standardization and consensus among laboratories. These aspects have been addressed by the only inter-laboratory study focused on molecular techniques for the diagnosis of histoplasmosis published to date (Buitrago et al., 2013). Seven PCR protocols (conventional and real time) were compared, with an overall sensitivity and specificity of 86 and 100% respectively. The results of this work led the authors to conclude that multicopy targets were the best option when designing an assay as they provide and increase in sensitivity without decreasing specificity; real time PCR proved to be more advantageous than conventional PCR. In contrast, one study limited to a small number of samples from a single laboratory showed a better sensitivity of nested PCR assays as compared to designs based on cycling-probe real-time PCR (Muraosa et al., 2016). Such differences highlight the need of collaborative networks to assess the diagnostic yield of different molecular assay designs for the diagnosis of histoplasmosis, particularly in areas of low prevalence.

## Future Directions and Conclusion

Education has proven to be an essential tool to increase the recognition of cases in endemic resource-constrained settings (Caceres et al., 2015), and also, it may be a future strategy to implement in non-endemic areas. Other pillars of utmost importance to effectively fight against histoplasmosis should be to consider it a notifiable infection, to quantify the real extent of the problem (Bongomin et al., 2019b; Staffolani et al., 2020), and to put into practice the use of rapid and reliable tools to detect and control potential environmental and animal sources of human infection (Cunningham et al., 2015; Gómez et al., 2018, 2019; Rothenburg et al., 2019).

Sadly, histoplasmosis is not classified as a neglected disease by organizations involved in Public Health, and published cases are thought to be just the tip of the iceberg. This hampers the development of affordable and accurate diagnostic tools. Efforts, however, are under way. A Colombian group is working on the development of an IGRA-based assay with promising preliminary results, and a huge potential for the diagnosis of subclinical infections regardless of the immune status of the host (Rubio-Carrasquilla et al., 2019); more results are awaited. A lateral flow device (LFD) for the detection of *Histoplasma* antigen in serum has been developed recently showing an excellent sensitivity, and extensive validation in non-progressive disseminated cases is expected (Cáceres et al., 2019). Molecular test might be part of the diagnostic armamentarium in settings where clinicians and laboratory personnel are not familiar with this pathogen. Reference laboratories from non-endemic regions with growing number of histoplasmosis cases are accumulating experience, and they are developing new assays that could also be of great help in areas of high endemicity.

Much remains to be done to improve the laboratory diagnosis of imported histoplasmosis. Efforts include extensive standardization and validation of already developed PCR-based techniques, and definition of the diagnostic yield in different types of samples and clinical settings. Initiatives to perform multicenter studies in non-endemic regions are being launched (Buitrago MJ, personal communication) to achieve a consensus on technical issues such as the best DNA extraction method or the most suitable targets, among others.

In conclusion, histoplasmosis is a primary fungal infection increasingly seen in non-endemic countries as a result of recreational travels and migratory movements. In receptor areas timely diagnosis is hampered by the lack of clinical awareness, and the scarcity of laboratory techniques able to provide accurate results with a short turn-around time regardless of the immune status of the host and the extent of the disease. Molecular techniques are seen as a suitable alternative for this purpose in areas of low prevalence. Much needed efforts to standardize such assays and to define their diagnostic yield are in progress. Molecular test promise to be of great help non-endemic areas, and as adjunctive tests for the laboratory diagnosis of histoplasmosis in areas of high endemicity.

## Author Contributions

MB and MM-G contributed equally to the design, elaboration, and review of this manuscript.

## Conflict of Interest

The authors declare that the research was conducted in the absence of any commercial or financial relationships that could be construed as a potential conflict of interest.

## Footnotes

1 https://www.who.int/medical_devices/publications/Standalone_document_v8.pdf
